# Orthogonal test design for optimization of the extraction of polysaccharide from *Paeonia sinjiangensis* K.Y. Pan

**DOI:** 10.4103/0973-1296.75874

**Published:** 2011

**Authors:** Shuge Tian, Xiaoying Zhou, Haiyan Gong, Xiuming Ma, Fan Zhang

**Affiliations:** 1 *Xinjiang Key Laboratory of Famous Prescription and Science of Formulas, XinJiang, China*; 2 *College of TCM, XinJiang, China*; 3 *College of Pharmacy, XinJiang, China*; 4 *College of Basic Medical, XinJiang Medical University, Urumqi - 830 011, XinJiang, China*

**Keywords:** Anthrone colorimetry, extraction, orthogonal experiment, *Paeonia sinjiangensis* K.Y. Pan, polysaccharide

## Abstract

**Background:**

*Paeonia sinjiangensis* K.Y. Pan is a perennial herb belonging to the ranunculaceae family and it is one of the most important crude drugs in traditional Chinese medicine. In this article, *Paeonia sinjiangensis* K.Y. Pan rich in polysaccharide is used as an experimental material.

**Materials and Method:**

Study the effects of proportion, temperature, times and time taken for the extraction yield of polysaccharide through a single-factor exploration. Then, through an orthogonal experiment (L_9_(3)^4^), it was investigated to get the best extraction conditions.

**Results:**

The results showed that the ratio of solvent to raw material, number of extractions and duration of extraction were the main variables that influenced the yields of extracts. The separation procedure of precipitation with alcohol and the purification from the removing proteins were deeply analyzed. Meanwhile the contents of polysaccharide were determined by anthrone colorimetry.

**Conclusion:**

The highest yield was obtained when the ratio of solvent to raw material, number of extractions, and duration of extraction were 8:1, 2, and 1.5 h, respectively. The content of soluble polysaccharide is 51.57%.

## INTRODUCTION

*Paeonia sinjiangensis* K.Y. Pan is a perennial herb belonging to the family Ranunculaceae. It is native to Xinjiang of China and is naturalized in the Altai mountain area, mostly in the western Xinjiang region. It is one of the most important crude drugs in traditional Chinese medicine, used as an anti-inflammatory, analgesic, and sedative agent. It is also frequently used as a herbal drug for its reputed medicinal properties, for example, as a remedy for diseases of women[1] and anti-hepatic fibrosis,[2] and as an anticoagulant, antithrombotic, antiatherosclerosis, and antitumor agent; to inhibit platelet and erythrocyte aggregation; and to protect the heart and the liver; and so on.[3] From the large amount of concerned literature, it was found that few reports have been published regarding the polysaccharides of this species and even less has been published regarding its acidic polysaccharide properties.[4] In a study by Tomoda *et al*., the authors demonstrated that an acidic polysaccharide, called peonan PA, was isolated from the root of *Paeonia lactiflora*. It was homogeneous on electrophoresis and gel chromatography, and the polysaccharide exhibited remarkable reticuloendothelial system-potentiating activity in a carbon clearance test and considerable anticomplementary activity. Recently, an article was published, concerning the chemical composition of *Paeonia anomala* subsp. *veitchii* (Paeoniaceae).[5] In recent years, a large number of articles have been published on the study of the contents of *paeoniflorin* using the extraction technology of *Radix paeoniae rubra*.[6–10] However, there is few literatures on polysaccharide of *P. sinjiangensis* K.Y. Pan. So the aim of this study was to carry out a more exhaustive analysis of *P. sinjiangensis* K.Y. Pan. Thus, the polysaccharide from *P. sinjiangensis* K.Y. Pan as the research object. The separation procedure of precipitation with ethanol and the purification by removing the proteins were deeply analyzed, and the contents of polysaccharide were determined by anthrone colorimetry.

## MATERIALS AND METHODS

### Materials

*P. sinjiangensis* K.Y. Pan plants were collected from the Altai mountain area of Xinjiang Province, China. Voucher specimens were deposited in Traditional Chinese Medicine College Museum of Chinese herbal samples of Xinjiang Medical University.

D-Glucose anhydrous (National Institute for The Control of Pharmaceutical and Biological Products, 110833), ethanol, chloroform, *n*-butanol, and sulfuric acid were obtained from Tianjin Reagent Co. (Tianjin, China). All other solvents and chemicals were analytically graded and purchased from Tianjin Fu-Yu Chemical Ltd., Co (Tianjin, China).

### Preparation of polysaccharide from *P. sinjiangensis* K.Y. Pan

*P. sinjiangensis* K.Y. Pan were homogenized in a blender to obtain a fine powder. The fine powder (5.0 g) was extracted with deionized water (water-powder (mL/g) ranging from 8:1 to 12:1), while the temperature of the water bath ranged from 65°C to 95°C and was kept steady. The water–powder slurry in the gas bath constant temperature oscillator was kept oscillating for a given time (duration of extraction ranging from 0.5 to 2.5 h) during the entire extraction process and the given number of extractions ranged from 1 to 3 times. The mixture was centrifuged (5 min, 3000 rpm, 25°C), and then the supernatant was separated from insoluble residue with filter paper. The extracts were then defatted by the method of Sevag, precipitated by the addition of ethanol to a final concentration of 75% (v/v), and the precipitates were collected by a centrifuge (5 min, 3000 rpm, 25°C), then solubilized in deionized water and lyophilized to get the crude polysaccharide.

### Preparation of standard curve

**Figure 1:**
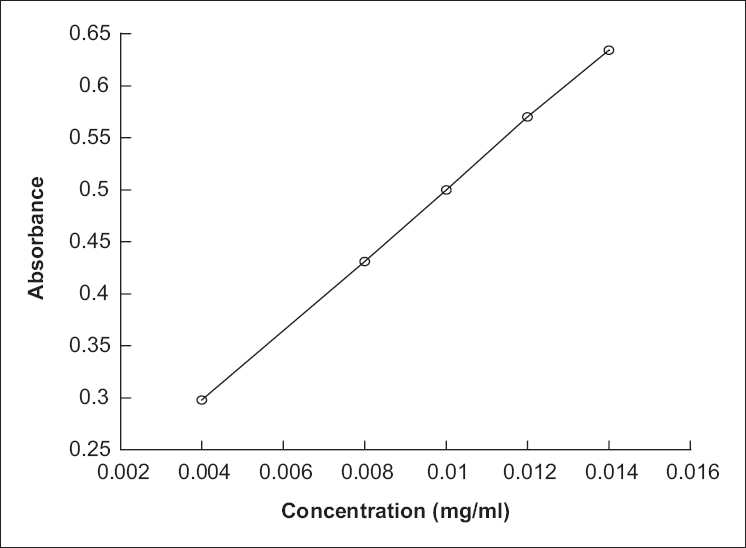
The standard curve of determination of glucose

Preparation of standard curve: 0.2, 0.4, 0.5, 0.6 and 0.7 mL glucose standard solutions were put into separate tubes, and diluted with water till 10 mL. Then 1 mL dilute solution and 0.2% 4 mL of anthrone and sulfuric acid, respectively, were poured into each tube. After fully mixing for 20 min, average Abs value of each tube was examined in 620 nm with 1 mL distilled water as contrast by using Cintra 40 UV–Vis spectrophotometer (GBC, Australia). The regression equation was Y = 33.77*x* + 0.1624 (*r* = 0.9999), linear range was 1.113–18.88 μg/mL [Figure 1 and Table 1].

**Table 1:** Absorbance value of glucose solution of various concentrations

Concentration (mg/mL)	0.004	0.008	0.01	0.012	0.014
Average Abs value	0.298	0.431	0.5	0.57	0.634

### Optimization of polysaccharide extraction

An orthogonal L_9_ (3)^4^ test design in the extraction mode was used for optimization of the extraction conditions. In the single-factor test, according to experimental methods, relative literatures, *et al*. to inspect single factor effect. Nine extractions were carried out at the ratios of solvent to raw material 8:1, 10:1, 12:1. Duration of extraction of 1.5, 2.0, and 2.5 h, number of extractions 1, 2, 3 on the basis of the single-factor test. Table 2 shows the experimental conditions for the extraction of polysaccharide from *P. sinjiangensis* K.Y. Pan.

**Table 2:** Factors and levels for orthogonal test

Variable	Level		
	1	2	3
Ratio of solvent to raw material	8:1	10:1	12:1
Number of extractions	1	2	3
Duration of extraction (h)	1.5	2	2.5

## RESULTS AND DISCUSSION

### Effect of the ratio of solvent to raw material on extraction yield of polysaccharide from *P. sinjiangensis* K.Y. Pan

**Figure 2:**
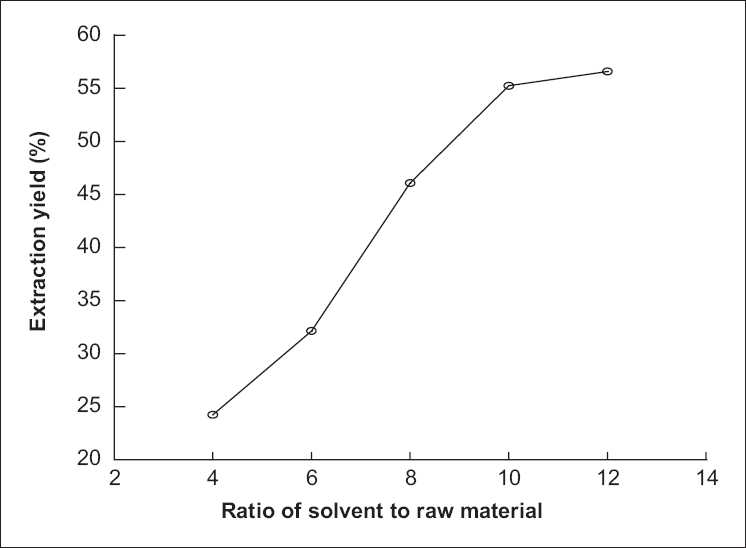
Effect of ratio of solvent to raw material on extraction yield of polysaccharide from *Paeonia sinjiangensis* K.Y. Pan

In this work, the effect of ratio of solvent to raw material on extraction yield of polysaccharide from *P. sinjiangensis* K.Y. Pan was investigated, and the results are listed in Figure 2. First, the other extraction conditions of polysaccharide from *P. sinjiangensis* K.Y. Pan, that is, duration of extraction, extraction temperature, and number of extractions, were fixed at 2 h, 85°C, 2, respectively, and the ratio of solvent to raw material was changed a little. As shown in Figure 1, the extraction yield of polysaccharide from *P. sinjiangensis* K.Y. Pan continued to increase with the increasing ratio of solvent to raw material and reached the peak value (55.26%) when the ratio of solvent to raw material was 10:1. However, when the ratio continued to increase, the extraction yield changed slowly.

### Effect of number of extractions on extraction yield of polysaccharide from *P. sinjiangensis* K.Y. Pan

**Figure 3:**
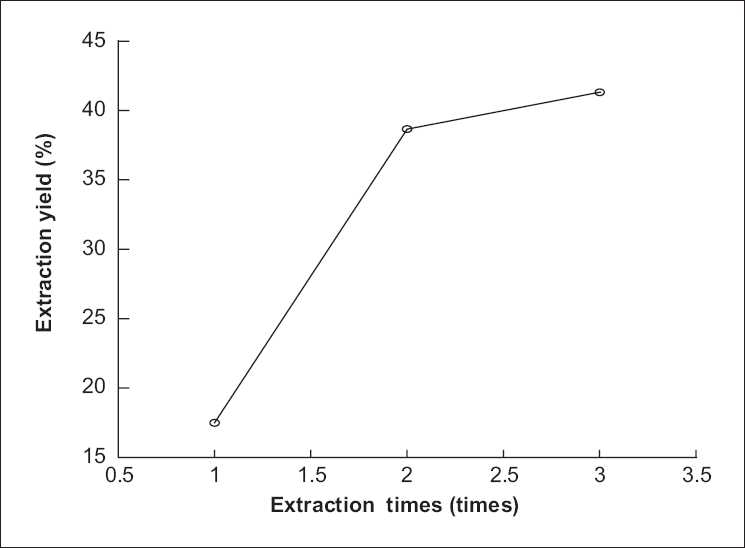
Effect of number of extractions on extraction yield of polysaccharide from *Paeonia sinjiangensis* K.Y. Pan

The effect of ratio of number of extractions on extraction yield of polysaccharide from *P. sinjiangensis* K.Y. Pan is shown in Figure 3. First, the number of extractions were set at 1, 2, and 3, while other extraction parameters were given as following: ratio of solvent to raw material 10:1, extraction temperature 85°C and duration of extraction 2 h. The extraction yield of the polysaccharide significantly increased from 17.50% to 38.67% as the number of extractions increased from 1 to 2 as showed in Figure 3. With increasing number of extractions from 2 to 3, the extraction yield of polysaccharide from *P. sinjiangensis* K.Y. Pan increased slowly. The maximum was 38.67% when the number of extractions was 2 [Figure 3].

### Effect of duration of extraction on extraction yield of polysaccharide from *P. sinjiangensis* K.Y. Pan

**Figure 4:**
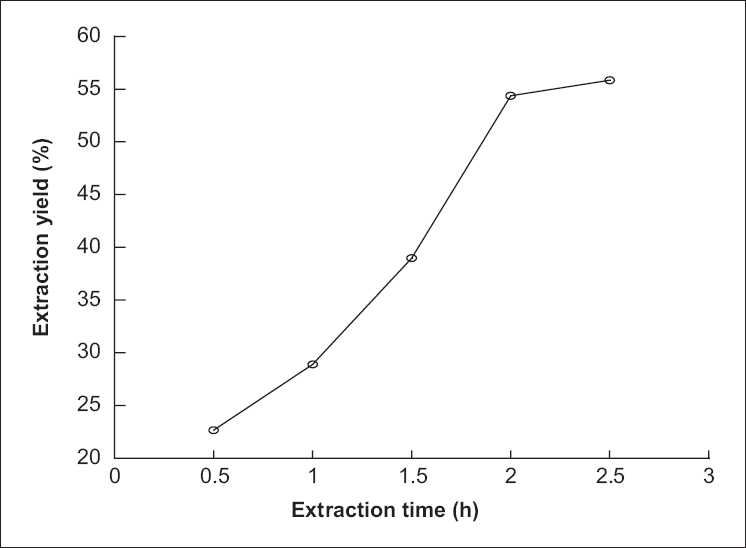
Effect of duration of extraction on extraction yield of polysaccharide from *Paeonia sinjiangensis* K.Y. Pan

Duration of extraction is another factor that would influence the extraction yield. The effect of duration of extraction on extraction yield of polysaccharide from *P. sinjiangensis* K.Y. Pan is shown in Figure 4. First, the duration of extraction was set at 0.5, 1.0, 1.5, 2.0, and 2.5 h, while the other extraction parameters were given as the following: ratio of solvent to raw material 10:1, extraction temperature 85°C and number of extractions 2. It could be found that by increasing the duration of extraction from 0.5 to 2 h, the extraction yield of polysaccharide from *P. sinjiangensis* K.Y. Pan increased from low to high and at 2 h to maximum, the extraction yield changed slowly.

### Effect of extraction temperature on extraction yield of polysaccharide from *P. sinjiangensis* K.Y. Pan

**Figure 5:**
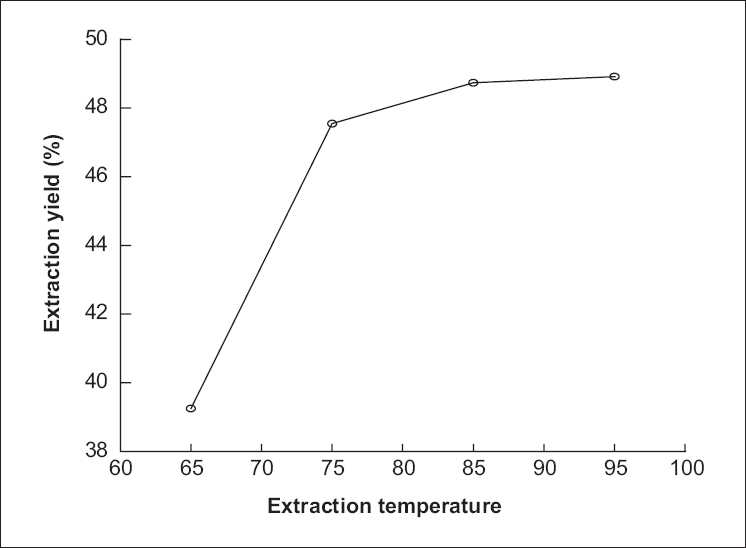
Effect of extraction temperature on extraction yield of polysaccharide from *Paeonia sinjiangensis* K.Y. Pan

In this work, the efficiencies of different extraction temperatures on the extraction yield of polysaccharide from *P. sinjiangensis* K.Y. Pan were investigated, and the results are listed in Figure 5. First, the other extraction conditions of polysaccharide from *P. sinjiangensis* K.Y. Pan, such as the ratio of solvent to raw material, duration of extraction, and number of extractions were fixed at 10:1, 2 h, and 2, respectively, and extraction temperature was slightly changed. As shown in Figure 5, the extraction yield of polysaccharide from *P. sinjiangensis* K.Y. Pan continued to increase with the increasing temperature and reached the peak value (48.74%) when extraction temperature was 85°C. The extraction yield of polysaccharide from *P. sinjiangensis* K.Y. Pan changed slowly after the extraction temperature exceeded 85°C.

### Optimization of the extraction parameters of polysaccharide from *P. sinjiangensis* K.Y. Pan

The first step in the extraction procedure of polysaccharide from *P. sinjiangensis* K.Y. Pan is to optimize the operating conditions to obtain an efficient extraction of the target compounds and avoid the co-extraction of the undesired compounds, such as fatty acids and their esters. Since various parameters potentially affect the extraction process, the optimization of the experimental conditions is a critical step in the development of a solvent extraction method. In fact, the ratio of solvent to raw material, number of extractions, and duration of extraction are generally considered to be the most important factors. Optimization of the suitable extraction conditions in the polysaccharide extraction can be carried out by using an experimental design. In the present study, all the selected factors were examined using an orthogonal L_9_ (3)^4^ test design. The total evaluation index was used to analyze by statistical method. The results of orthogonal test and extreme difference analysis are presented in Tables 3 and 4. The analysis of variance was performed by statistical software SPSS 12.0 (SPSS Inc.) and the result is listed in Table 4.

**Table 3:** Analysis of L_9_(3)^4^ test results

No.	A, ratio of solvent to raw material	B, number of extractions	C, duration of extraction (h)	D, blank factor	Extraction yield (%)
1	1	1	1	1	51.43
2	1	2	2	2	50.64
3	1	3	3	3	51.57
4	2	1	2	3	48.14
5	2	2	3	1	49.55
6	2	3	1	2	49.26
7	3	1	3	2	41.93
8	3	2	1	3	44.89
9	3	3	2	1	42.45
*K*_1_	153.64	141.5	145.58	143.31	
*K*_2_	146.95	145.08	141.23	141.83	
*K*_3_	129.07	143.08	143.05	144.6	
R	8.12	1.19	1.45	0.92	

**Table 4:** Variance analysis results

Variation sources	SS	*V*	MS	*F*	*P*
A	105.69	2	52.85	81.99	<0.05
B	2.14	2	1.07	1.66	>0.05
C	3.18	2	1.59	2.47	>0.05
D	1.29	2	0.64	1	
Error	1.29	2	0.64		

SS; *V*; MS; *F*_0.05(2,2)_ = 19

The extract obtained from each test in the polysaccharide extraction was weighed and quantitatively analyzed and then the extraction yields of the crude extract and each compound were calculated. The results of experiments presented in Table 3 indicate that the maximum extraction yield of the crude extract was 51.57%. However, we could not select the best extraction conditions only based on these outcomes in Table 3, and a further orthogonal analysis was warranted. Thus, the K, *k*, and R values were calculated and listed in Table 3. As seen from Table 3, we can find that the influence to the mean extraction yields of the compounds decreases in the order: A > C > B > D according to the *R* values. The ratio of solvent to raw material was found to be the most important determinant of the yield. In other words, the maximum yield of the polysaccharide was obtained when ratio of solvent to raw material, number of extractions, and duration of extraction were 8:1, 2, and 1.5 h, respectively.

## CONCLUSION

From the experiment, we can conclude that the ratio of solvent to raw material was the most important determinant of the yield. The highest yield was obtained when ratio of solvent to raw material, number of extractions, and duration of extraction were 8:1, 2, and 1.5 h, respectively. The content of soluble polysaccharide is 51.57%. In this article, *P. sinjiangensis* K.Y. Pan-enriched polysaccharide is used as an experimental material to study the effect of proportion, temperature, times, and time on extraction yield of polysaccharide through a single-factor exploration. The results showed that the ratio of solvent to raw material, number of extractions, and duration of extraction were the main variables that influenced the yields of the extracts and were investigated through an orthogonal experiment (L_9_ (3)^4^) to get the best extraction conditions. The separation procedure of precipitation with alcohol and the purification by removing the proteins were deeply analyzed. Meanwhile, the contents of polysaccharide were determined by anthrone colorimetry. Besides, from the large amount of concerned literature, it was found that few reports have been published regarding the polysaccharide from *P. sinjiangensis* K.Y. Pan. However, the article has done the preliminary research of the polysaccharide from *P. sinjiangensis* K.Y. Pan. So it can be used as a reference to do further research of the polysaccharide from *P. sinjiangensis* K.Y. Pan for the other research works.
